# Eosinophils, Stroke-Associated Pneumonia, and Outcome After Mechanical Thrombectomy for Acute Ischemic Stroke

**DOI:** 10.3389/fnagi.2022.830858

**Published:** 2022-05-23

**Authors:** Zhiliang Guo, Jie Hou, Shuai Yu, Hang Zhang, Shuhong Yu, Huaishun Wang, Jiaping Xu, Shoujiang You, Zhichao Huang, Guodong Xiao, Yongjun Cao, Chun-Feng Liu

**Affiliations:** ^1^Department of Neurology and Suzhou Clinical Research Center of Neurological Disease, The Second Affiliated Hospital of Soochow University, Suzhou, China; ^2^Department of Encephalopathy, Suzhou Integrated Traditional Chinese and Western Medicine Hospital, Suzhou, China

**Keywords:** eosinophils, stroke-associated pneumonia, outcome, immunosuppression, mechanical thrombectomy

## Abstract

**Background:**

Eosinophils contribute to antibacterial defense by releasing mitochondrial DNA, which are decreased in patients with acute ischemic stroke (AIS). However, the impact of eosinophils on stroke-associated pneumonia (SAP) among patients with AIS remains unclear. Moreover, whether SAP is in the path of the association between eosinophils and clinical outcomes also remains unclear. We aimed to assess the relationships between eosinophils, SAP, and clinical outcome after mechanical thrombectomy in patients with AIS.

**Methods:**

A total of 328 consecutive patients with AIS who underwent mechanical thrombectomy between May 2017 and March 2021 were analyzed. Their baseline data and peripheral eosinophil counts were recorded on admission. Regression analysis was used to assess the effect of eosinophils on SAP, and its effect on poor outcome is defined as a modified Rankin Scale score of 3–6 at month 3 after admission. Mediation analysis was utilized to assess the proportion of the total effect of SAP on the association between eosinophils and poor outcomes.

**Results:**

Multivariate analysis revealed that eosinophils was independently associated with SAP after adjusting for potential confounders (odds ratio, 0.00; 95% CI, 0.00–0.38; *P* = 0.0267), which are consistent with the result of eosinophils (dichotomous) as a categorical variable (odds ratio, 0.54; 95% CI, 0.31–0.96; *P* = 0.0342). A non-linear relationship was detected between eosinophils and SAP, whose inflection point was 0.06. Subgroup analyses further confirmed these associations. Eosinophils were also associated with poor outcomes (odds ratio, 0.00; 95% CI, 0.00–0.14; *P* = 0.0124). Additionally, mediation analysis found that SAP partially mediated the negative relationship between eosinophils and poor outcome (indirect effect = −0.169; 95% CI:−0.339 –−0.040, *P* < 0.001).

**Conclusion:**

Our findings suggested that a lower eosinophil level was associated with higher SAP and poorer outcome, and SAP might play an important effect in the association between eosinophils and poor outcomes.

## Introduction

Eosinophils can regulate the innate immune responses by facilitating the resolution of inflammation, which play important roles in the pathogenesis of acute ischemic stroke (AIS) (Jucevičiute et al., 2019; Yu et al., 2021). Recent studies have discovered that stroke triggers an acute decrease in circulating eosinophil counts, and eosinophils are independently associated with the severity of the stroke and functional outcome in patients with AIS (Guo et al., 2015; Zhao et al., 2019). The underlying mechanisms explaining the link between eosinophils and functional outcomes are yet unclear, but possible and yet unexplored pathways are through stroke-induced immunosuppression and subsequent complications (Guo et al., 2015; Zhao et al., 2019).

Stroke-associated pneumonia (SAP) is a major cause of poor functional outcomes after stroke (Faura et al., 2021). The mechanisms of SAP seem to be related to stroke-induced immunosuppression, which include a decrease of the natural killer (NK) cells and lymphocyte counts, a shift from a lymphocyte phenotype T-helper 1 to a T-helper 2 phenotype, and an impairment of the defense mechanisms of monocytes and neutrophils (Faura et al., 2021). Moreover, eosinophils have also been shown to mediate immune responses by the catapult-like release of mitochondrial DNA (Yousefi et al., 2008). The mitochondrial DNA and the granule proteins released by eosinophils form extracellular structures that can bind and kill bacteria, which contributes to antibacterial defense (Yousefi et al., 2008). These data suggest that the decrease of eosinophils after stroke enhances SAP due to a decrease of the antimicrobial defense, thereby contributing to poor outcomes (Yousefi et al., 2008; Faura et al., 2021). In this view, it is plausible to assume that increased SAP may be in the path of the association between decreased eosinophils and poor outcomes. However, such a relationship has not been fully understood.

We aimed to assess the relationships between eosinophils, SAP, and clinical outcomes after mechanical thrombectomy in patients with AIS. In this study, we obtained the following three new observations: first, revealing the relationship between eosinophils and SAP; second, proving the correlation between low eosinophil levels and poor outcomes in patients with stroke treated with mechanical thrombectomy; and finally, SAP may partially mediate the negative relationship between eosinophils and poor outcomes. We expect all these results to provide novel ideas for uncovering the mechanisms of stroke-induced immunosuppression and SAP.

## Methods

The data that support the findings of this study are available from the corresponding author on reasonable request.

### Study Population

Patients with consecutive AIS experiencing endovascular treatment admitted to the Department of Neurology at our hospital from May 2017 to March 2021 were prospectively recruited. For retrospectively analyzing the relationships between eosinophils, SAP, and functional outcomes in the prospectively collected cohort of patients with AIS treated with endovascular treatment, the inclusion criteria for the current analysis were as follows: (1) age ≥18 years; (2) clinical diagnosis of AIS with the anterior or posterior circulation of large vessel occlusion; and (3) underwent first-line treatment with direct aspiration, stent retriever, or a combination of a stent retriever and local aspiration catheter. The exclusion criteria were as follows: (1) patients treated with intra-arterial thrombolysis only (56 patients); (2) patients with asthma, eosinophilic esophagitis, hypereosinophilic syndrome, evidence of active infection, chronic inflammatory, autoimmune diseases, steroid therapy, cancer, hematologic disease, severe hepatic, or renal dysfunction (4 patients); (3) patients with unavailable complete blood cell count, with incomplete medical records, or who are lost to follow-up (9 patients). Finally, 328 patients with consecutive AIS were included in this study (flowchart of participant selection: Figure I in Appendix A). The study protocol was approved by the Ethics Committee of the Second Affiliated Hospital of Soochow University, and all patients or their relatives gave written informed consent.

### Clinical Protocol and Laboratory Tests

Medical history including demographics (age, female), potential stroke risk factors (atrial fibrillation [AF], hypertension, diabetes, hyperlipidemia, smoking, and drinker status), presence of dysphagia (based on the water swallow test) (Zhu et al., 2020), stroke etiology, stroke severity, admission of the Alberta Stroke Program Early CT Score (ASPECTS), pretreatment with intravenous thrombolysis (IVT), premorbid modified Rankin Scale (mRS) score, occlusion site, the status of the collateral circulation, symptom onset or when last seen well to reperfusion time, blood tests, 12-lead electrocardiogram, and chest radiography/CT were performed at admission. Additionally, stroke risk factors were defined according to previously published criteria (Wang et al., 2014). Dysphagia was evaluated using bedside swallowing tests performed at our hospital within the 1st day, which were confirmed by the specialized nurses of stroke if the water swallow test score was ≥ 2 or experiencing nasogastric tube intubation treatment. The etiologic subtypes and stroke severity were recorded as described in previous studies (Xiao et al., 2020; Zhu et al., 2020). The status of the collateral circulation before thrombectomy was evaluated using the American Society of Interventional and Therapeutic Neuroradiology/Society of Interventional Radiology scale (Higashida et al., 2003). Reperfusion status was graded in the final angiogram according to the modified Thrombolysis in Cerebral Infarction (mTICI) score, with successful recanalization defined as a score of 2b or 3 (Xiao et al., 2020; Zhu et al., 2020). Peripheral venous blood samples were obtained on admission for the measurement of eosinophil levels. The poor outcome was defined as a modified Rankin Scale score of 3–6 at 3 months after admission (Xiao et al., 2020; Zhu et al., 2020).

### Assessment of SAP

Diagnosis of SAP was according to the consensus given in previous studies (Smith et al., 2015; Zhu et al., 2020). The diagnosis of SAP met the criteria as follows: at least one of the following: (1) fever (>38°C) with no other recognized cause, (2) leukopenia or leukocytosis, and (3) altered mental status with no other recognized cause for adults ≥70 years old; and at least 2 of the following: (1) new onset of purulent sputum, change in the character of sputum over a 24 h period, increased respiratory secretions, or increased suctioning requirements, (2) new onset or worsening cough, or respiratory rate, (3) rales, crackles, or bronchial breath sounds, and (4) worsening gas exchange; and ≥2 serial chest imaging with at least 1 of the following: new or progressive and persistent infiltrate, consolidation, or cavitation. In patients without underlying pulmonary or cardiac disease, one definitive chest imaging is acceptable (Smith et al., 2015; Zhu et al., 2020). Some typical imaging manifestations of the CT are shown in Figure II in Appendix A.

### Statistical Analysis

All analyses were performed using EmpowerStats (http://www.empowerstats.com, X&Y Solutions, Inc., Boston, MA) and the statistical software package R (http://www.R-project.org, The R Foundation). Two-sided values of *P* < 0.05 were considered statistically significant. The total procedure of statistical analysis was divided into four steps (Guo et al., 2021; Yu et al., 2021).

First, the baseline characteristics of study participants were presented according to the dichotomous eosinophils. The chi-square test or the Mann–Whitney *U* test was used to determine any significant differences between groups according to the dichotomous of eosinophils.

Second, we used a univariate regression model, multivariable regression analysis, generalized additive models, and subgroup analyses to explore the relationships between eosinophils and SAP. (1) We used a univariate regression model to evaluate the associations between eosinophils and SAP in patients with AIS experiencing mechanical thrombectomy. For multivariate analysis, we first included age and women (model 1) and then included variables in the final models if they were significantly associated with SAP (*P* < 0.10) or changed the estimates of eosinophils on SAP by more than 10% (model 2; age, hyperlipidemia, baseline National Institutes of Health Stroke Scale (NIHSS), ASPECTS, occluded artery, dysphagia, stroke etiology, and collateral score). Tables II–V in Appendix A show the associations of each confounder with the outcomes of interest (Guo et al., 2021; Yu et al., 2021). (2) We used generalized additive models to identify the non-linear relationships because eosinophils were a continuous variable. If a non-linear relationship was observed, a two-piecewise linear regression model was used to calculate the threshold effect of the eosinophils on SAP in terms of the smoothing plot. When the ratio between eosinophils and SAP appeared obvious in a smoothed curve, the recursive method automatically calculates the inflection point, where the maximum model likelihood will be used (Guo et al., 2021; Yu et al., 2021). (3) We conducted subgroup analyses to assess the robustness of the association between low eosinophils and SAP using stratified regression models. The modifications and interactions between eosinophils and subgroup variables on the SAP were tested by likelihood ratio tests (Guo et al., 2021; Yu et al., 2021).

Third, the association between eosinophils and poor outcomes was also assessed using a univariate regression model, multivariable regression analysis, generalized additive models, and subgroup analyses.

Fourth, using mediation analyses, we evaluated whether SAP mediated the relationship between eosinophils and the functional outcome after controlling for potential confounders. We simultaneously considered the direct, indirect, and total effects of predictors on outcomes through mediators (VanderWeele, 2016).

## Results

### Baseline Characteristics of Patients

In addition to the excluded patients having less AF and different stroke etiologies, most of the baseline characteristics were balanced between patients included and patients excluded (Table I in Appendix A). A total of 328 patients with AIS experiencing mechanical thrombectomy were included in this study, and the median age was 68 years. The main baseline characteristics of study participants according to dichotomous eosinophils are presented in Table 1. The participants with lower eosinophil values had higher baseline NIHSS, mRS score, and proportion of dysphagia but lower ASPECTS and proportion of smoking. These patients presented different occluded arteries and also were more likely to have a higher risk of developing SAP and poor outcome (Table 1).

**Table 1:** Baseline characteristics of study participants according to the eosinophil level.

**Characteristics**	**Low eosinophil**	**High eosinophil**	***P*-Value**
	**level**	**level**	
No. of patients	141	187	
Age, y; median (IQR)	68.00 (60.00–77.00)	67.00 (55.50–74.00)	0.187
Female, *n* (%)	69 (48.94%)	74 (39.57%)	0.090
Atrial fibrillation, *n* (%)	67 (47.52%)	80 (42.78%)	0.393
Hypertension, *n* (%)	97 (68.79%)	128 (68.45%)	0.947
Diabetes, *n* (%)	27 (19.15%)	34 (18.18%)	0.824
Hyperlipidemia, *n* (%)	43 (30.50%)	72 (38.50%)	0.132
History of stroke, *n* (%)	26 (18.44%)	27 (14.44%)	0.330
Smoking, *n* (%)	33 (23.40%)	67 (35.83%)	0.016
Drinking, *n* (%)	27 (19.15%)	46 (24.60%)	0.240
Baseline NIHSS, median (IQR)	18.00 (14.00–21.00)	15.00 (12.00–18.00)	<0.001
ASPECTS, median (IQR)	7.00 (6.00–7.00)	7.00 (7.00–8.00)	0.007
**Occluded artery**, ***n*** **(%)**			0.005
ICA	40 (28.37%)	30 (16.04%)	
M1 of the MCA	73 (51.77%)	126 (67.38%)	
Posterior circulation	21 (14.89%)	16 (8.56%)	
Others	7 (4.96%)	15 (8.02%)	
IVT, *n* (%)	43 (30.50%)	60 (32.09%)	0.759
Dysphagia, *n* (%)	95 (67.38%)	86 (45.99%)	<0.001
Premorbid mRS, median (IQR)	0.00 (0.00–0.00)	0.00 (0.00–0.00)	0.788
**Stroke etiology**, ***n*** **(%)**			0.825
LAA	59 (41.84%)	84 (44.92%)	
Cardioembolic	75 (53.19%)	93 (49.73%)	
Others	7 (4.96%)	10 (5.35%)	
Collateral score, median (IQR)	0.00 (0.00–1.00)	0.00 (0.00–2.00)	0.079
OTR, median (IQR), min	352.50 (290.50–440.50)	335.50 (264.25–424.50)	0.053
Number of passes, median (IQR)	2.00 (1.00–3.00)	2.00 (1.00–2.50)	0.062
mTICI score 2b or 3, *n* (%)	122 (86.52%)	167 (89.30%)	0.441
SAP, *n* (%)	92 (65.25%)	80 (42.78%)	<0.001
mRS score	4.00 (3.00–6.00)	2.00 (1.00–4.00)	<0.001
Poor outcome	119 (84.40%)	92 (49.20%)	<0.001

*ASPECTS, Alberta stroke program early CT score; IQR, interquartile range; ICA, internal carotid artery; IVT, intravenous thrombolysis; MCA, middle cerebral artery; mRS, modified rankin scale; mTICI, modified thrombolysis in cerebral infarction; NIHSS, national institutes of health stroke scale; OTR, onset to reperfusion time; posterior circulation, including basilar artery and intracranial part of the vertebral artery; SAP, stroke-associated pneumonia*.

### The Univariate and Multivariate Analyses Exploring the Relationships Between Eosinophils and SAP

The results of the univariate analysis showed that age, baseline NIHSS, posterior circulation occlusion (basilar artery and intracranial part of the vertebral artery), and dysphagia were positively correlated with SAP, whereas ASPECTS, other stroke etiologies, and the collateral score were negatively associated with SAP (Table III in Appendix A).

Table 2 summarizes the results of multivariable linear regression analysis. The eosinophils as a continuous variable were independently associated with SAP with an adjusted odds ratio (OR) of 0.00 (95% confidence interval (CI), 0.00–0.02; *P* = 0.0014) after adjustment for age and women (model 1) and 0.00 (95% CI, 0.00–0.38; *P* = 0.0267) after adjustment for all potential covariates (model 2). For the purpose of sensitivity analysis, we converted the eosinophils into categorical variables by dichotomous, and the OR (95% CI) of SAP for the participants with higher eosinophils was 0.54 (0.31–0.96) compared with patients in the lower percentiles of eosinophils.

**Table 2:** Relationship between eosinophils and the SAP/functional outcome among patients with acute ischemic stroke in different models.

**Variable**	**Non-Adjusted model**		**Model 1**		**Model 2**	
	**β/OR (95% CI)**	***P-*Value**	**β/OR (95% CI)**	***P-*Value**	**β/OR (95% CI)**	***P-*Value**
SAP	0.00 (0.00, 0.01)	0.0010	0.00 (0.00, 0.02)	0.0014	0.00 (0.00, 0.38)	0.0267
mRS	−12.43 (−17.26, −7.60)	<0.0001	−11.30 (−15.89, −6.71)	<0.0001	−5.36 (−9.24, −1.48)	0.0072
Poor outcome (mRS score 3–6)	0.00 (0.00, 0.00)	<0.0001	0.00 (0.00, 0.00)	0.0001	0.00 (0.00, 0.14)	0.0124

*Non-adjusted model: we did not adjust other covariates*.

*Model 1: we adjusted for age and female gender*.

*Model 2: we adjusted variables that were significantly associated with outcomes of interest (P < 0.10) or changed the estimates of eosinophils on outcomes of interest by more than 10% (Tables II–V in Appendix A)*.

*mRS, modified Rankin Scale; OR, odds ratio; SAP, stroke-associated pneumonia*.

### The Analyses of the Non-Linear Relationship Between Eosinophils and SAP

**Figure 1:**
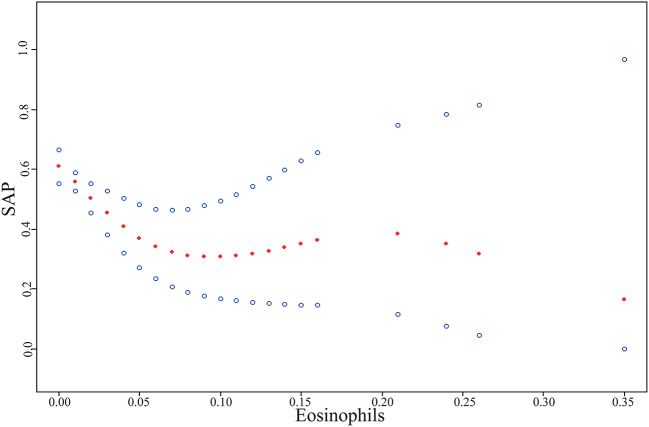
The non-linear relationship between eosinophils and stroke-associated pneumonia (SAP) in patients with acute ischemic stroke experiencing mechanical thrombectomy. A non-linear relationship between them was detected after adjusting for age, hyperlipidemia, baseline National Institutes of Health Stroke Scale (NIHSS), Alberta Stroke Program Early CT Score (ASPECTS), occluded artery, dysphagia, stroke etiology, and collateral score.

In this study, we analyzed the non-linear relationship between eosinophils and SAP (Figure 1). The result of the smooth curve showed that the relationship between eosinophils and SAP was non-linear after adjustment for all potential covariates (*P* = 0.031). We compared the linear regression model (fitting the relationship between eosinophils and SAP by a linear) and the two-piecewise linear regression model (fitting the relationship between eosinophils and SAP by a curve) (Table 3). The *P*-value for the log-likelihood ratio test is 0.020, which is less than 0.05. This result indicates that the two-piecewise linear regression model should be used to fit the relationship between eosinophils and SAP. By using a two-piecewise linear regression model, we calculated that the inflection point was 0.06. On the left of inflection point, the effect size was 0.0000 (95% CI: 0.0000–0.0001, *P* = 0.0021). However, on the right side of the inflection point, we did not observe a significant association between eosinophils and SAP (19.1626, 95% CI: 0.0007-561818.8810, *P* = 0.5736; Table 3). Moreover, subgroup analyses further confirmed these associations between eosinophils and SAP. As shown in Table VI in Appendix A, the test of interactions was not statistically significant for age, women, atrial fibrillation, hypertension, diabetes, hyperlipidemia, history of stroke, smoking, drinking, baseline NIHSS, IV thrombolysis, and dysphagia (*P*-values for interaction were larger than 0.05).

**Table 3:** The results of the two-piecewise linear regression model.

**Outcome**	**Effect size OR (95%CI)**	***P*-Value**
**Inflection point of eosinophils**
<0.06	0.0000 (0.0000, 0.0001)	0.0021
≥0.06	19.1626 (0.0007, 561818.8810)	0.5736
*P* for log likelihood ratio test	0.020	

*Effect: eosinophils; cause: SAP; adjusted: age, hyperlipidemia, baseline NIHSS, ASPECTS, occluded artery, dysphagia, stroke etiology, and collateral score*.

*OR, odds ratio; SAP, stroke-associated pneumonia; NIHSS, National Institutes of Health Stroke Scale; ASPECTS, Alberta Stroke Program Early CT Score*.

### The Association Between Eosinophils and Functional Outcome

In terms of the relationship between eosinophils and functional outcomes, the generalized linear analysis revealed that eosinophils were independently associated with lower mRS scores after adjusting potential confounders (β,−5.36; 95% CI,−9.24–1.48; *P* = 0.0072). We used eosinophils (dichotomous) as a categorical variable in this study and found similar results (β,−0.80; 95% CI,−1.15–0.44; *P* < 0.001). Multivariate logistic regression analysis also revealed that eosinophils were independently associated with poor outcome (mRS score 3-6) after adjusting potential confounders (as a continuous variable: OR, 0.00; 95% CI, 0.00-0.14; *P* = 0.0124; as a categorical variable: OR, 0.20; 95% CI, 0.09-0.45; *P* = 0.0001. Table 2 and Table VII in Appendix A). The non-linear relationships were also found between eosinophils and functional outcomes in the generalized additive models (*P* = 0.0013 for mRS score; *P* = 0.0174 for poor outcome. Figures III, IV in Appendix A). However, there was no statistically significant inflection point in the threshold effect analyses (*p*-value for the log-likelihood ratio test is 1.000 for both the mRS score and poor outcomes). Subgroup analyses also further confirmed these associations between eosinophils and functional outcomes (Table IX in Appendix A).

### Mediation Analysis for Functional Outcome

**Figure 2:**
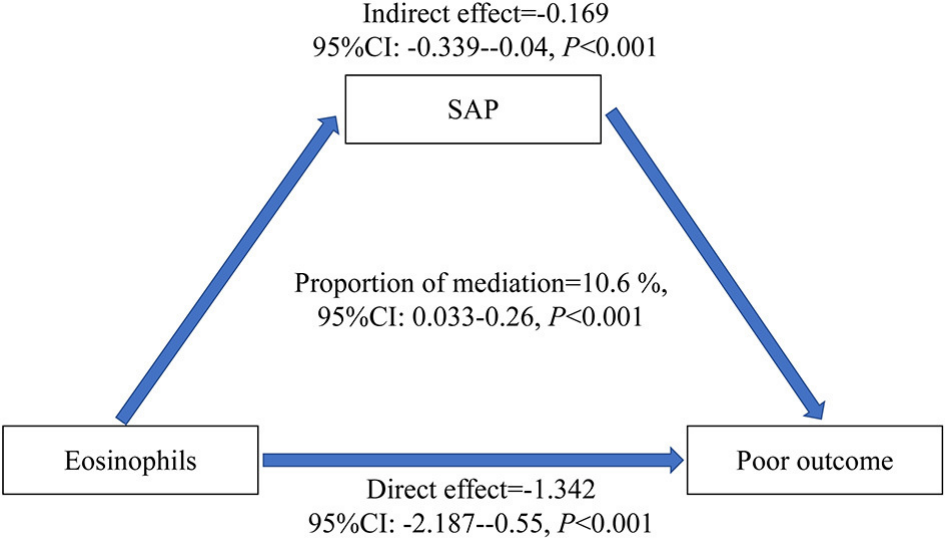
Schematic diagram of mediation analyses for functional outcome. The eosinophils were entered as predictors. SAP was entered as a mediator.

We constructed a hypothetical model of relationships among eosinophils, SAP, and functional outcome. Our results indicated that SAP partially mediated the relationship between eosinophils and poor outcomes (Figure 2). Additionally, the proportion of the total effect of eosinophils on the poor outcome mediated by SAP was 10.6% (95% CI, 3.3%−26%). The direct effect of eosinophils on the poor outcome (total effect minus indirect effect) was still statistically significant (*P* < 0.001) after removing the effect mediated by SAP.

## Discussion

Endovascular treatment is clinically effective and cost-saving in comparison to usual care alone in patients with AIS. SAP is one of the most common complications following AIS, which increases the risk of mortality and worsens the functional outcomes (Zhu et al., 2020; Faura et al., 2021; van den Berg et al., 2021; Zhang et al., 2021). The purpose of the present study was to assess the relationships between eosinophils, SAP, and functional outcome after mechanical thrombectomy in patients with AIS. In addition, this study showed that the decreased eosinophil level was associated with an increased risk of SAP. In addition, there existed an association between eosinophils and functional outcomes, which was in line with previous studies (Zhao et al., 2019; Semerano et al., 2020; Yu et al., 2021). More importantly, this study shows the significant role of SAP in this association. These results might provide novel ideas for uncovering the mechanisms of stroke-induced immunosuppression and SAP. Additionally, early detection of decreased eosinophils and the timely and effective management of SAP might improve functional outcomes after mechanical thrombectomy.

Many risk factors such as age, diabetes, stroke severity, and dysphagia have been identified for SAP already (Zhu et al., 2020; Faura et al., 2021; Zhang et al., 2021). To the best of our knowledge, this is the first study that investigated eosinophils specifically in relation to SAP and found that decreased eosinophils were associated with an increased SAP (OR, 0.00; 95% CI, 0.00–0.38; *P* = 0.0267). The mechanisms underlying these observations are not well established, but stroke-induced immunosuppression may play a key role in the development of SAP (Guo et al., 2015; Faura et al., 2021). A recent study revealed that ischemic stroke reduces the percentage of eosinophils in the lungs (Farris et al., 2019). The alteration of eosinophils coincides with a significant reduction in the levels of multiple chemokines and cytokines in the lungs, including CCL3, CCL5, CCL17, CCL22, CXCL5, CXCL10, IL-1β, TNF-α, IFN-γ, IL-17A, and IL-27 (Farris et al., 2019). Most of these chemokines and cytokines, such as CCL5, CCL22, IL-1β, and TNF-α, are critical to “pre-condition” the lungs to become invulnerable to bacterial infections or promote bacterial clearance during lung infections (Farris et al., 2019). These suggest that ischemic stroke creates an immunosuppression milieu in the lungs by decreasing the production of multiple proinflammatory chemokines and cytokines. Moreover, the release of mitochondrial DNA and the granule proteins by eosinophils forms extracellular structures that are able to bind and kill bacteria, thus contributing to antibacterial defense (Yousefi et al., 2008; Farris et al., 2019). These data suggest that the mechanisms underlying the correlation between decreased eosinophils and increased SAP seem to be related to the decrease in antimicrobial defense (Yousefi et al., 2008; Farris et al., 2019).

In terms of the relationship between eosinophils and functional outcomes, previous studies reported that stroke triggered an acute decrease in circulating eosinophil counts, and eosinophils were independently associated with the severity of the stroke and functional outcomes in patients with AIS (Zhao et al., 2019; Semerano et al., 2020; Yang et al., 2021; Yu et al., 2021). However, there are some flaws in their studies; such as not adjusting for NIHSS, the retrospective study design, or the relatively small sample size (Zhao et al., 2019; Semerano et al., 2020; Yang et al., 2021; Yu et al., 2021); and almost no research has been performed on patients with stroke treated with mechanical thrombectomy who usually have higher NIHSS. Therefore, further studies are needed to explore whether there exists an association between eosinophils and functional outcomes in patients with stroke treated with mechanical thrombectomy. In this study, we found a negative correlation between eosinophils and functional outcomes after mechanical thrombectomy in subjects with AIS. Given that the percentage of eosinophils was negatively correlated with infarct volume and eosinopenia had the potential to predict the severity of AIS (Hori et al., 2016; Zhao et al., 2019; Yu et al., 2021), we performed the collinearity screening and found that there was no collinearity between eosinophils and NIHSS (Table II in Appendix A). Moreover, we found that the significant association of eosinophils with poor outcomes was independent of the baseline NIHSS, especially in participants with stroke with relatively low NIHSS (NIHSS score <16: OR, 0.00; 95% CI, 0.00–0.01; *P* = 0.0068) in the multivariate analysis. These results suggested that we should apply eosinophils for the prediction of poor outcomes in participants with relatively low NIHSS. The reason why the effect sizes of eosinophils on poor outcomes showed differences in different NIHSS scores remains unclear. We hypothesized that the complications of patients with severe stroke and higher NIHSS itself might cause some disruptions to the relationship between eosinophils and poor outcomes. Further studies are needed to test this hypothesis.

The correlation between eosinophils and functional outcomes might be driven by both immunosuppressive and neuroprotective pathways. Eosinophils were associated with both SAP and functional outcome. SAP partially mediated the relationship between decreased eosinophil levels and poor outcomes. These data suggest that the stroke-induced immunosuppression and subsequent SAP complications may be one of the mechanisms of the correlation between eosinophils and functional outcomes. In addition, we found that the significant association of eosinophils with poor outcomes was independent of SAP in the multivariate analysis (mRS: β,−4.87; 95% CI,−8.77–0.97; *P* = 0.050; poor outcome: OR, 0.00; 95% CI, 0.00-0.39; *P* = 0.0269. Table X in Appendix A). These results suggested that eosinophils had an additional prognostic value when SAP was considered, and eosinophils might contribute to poor outcomes besides SAP, such as neuroprotection. Eosinophils can secrete vascular endothelial growth factors and multiple chemokines (Davoine and Lacy, 2014; Zierath et al., 2018; Yu et al., 2021). IL-4 and IL-13 secreted by eosinophils are capable of inducing the activation of the M2 phenotype microglia, which possess neuroprotective properties by facilitating the resolution of inflammation. In addition, vascular endothelial growth factors might be neuroprotective by the modulation of angiogenesis (Davoine and Lacy, 2014; Zierath et al., 2018; Yu et al., 2021). Alternatively, it is possible that decreased eosinophils might contribute to more SAP and less neuroprotection, which in turn lead to poor outcomes (Figure VI in Appendix A). To test our hypotheses, further studies with specificity for animal studies are needed.

The main strength of our study is that we provided a comprehensive study with the models fitted to assess the effect of SAP on the association between eosinophils and functional outcomes. Nonetheless, this study has some limitations. First, the eosinophil levels were only measured at a single point; therefore, results may vary due to possible rapid change of its values after the onset of symptoms (Guo et al., 2016; Yang et al., 2021). Second, we neither explored the mechanisms by which eosinophils affected the immunosuppressive and neuroprotective pathways or investigated what factors regulated the changes of eosinophils after ischemic strokes in animal studies. These are going to be the focus of our next study, especially exploring the role of eosinophils in SAP and its mechanism. Third, the cohort in this study represents a subgroup of patients with stroke due to large vessel occlusions who underwent thrombectomy; thus, the results reported in this study are not generalizable to the whole population of patients with stroke. In addition, the proportion of patients experiencing general anesthesia was so small that the baseline data of general anesthesia were not further included in this study. Nevertheless, we found that the significant association of eosinophils with SAP/poor outcome was independent of general anesthesia in the multivariate analysis (SAP: OR, 0.00; 95% CI, 0.00-0.38; *P* = 0.0263; poor outcome: OR, 0.00; 95% CI, 0.00-0.10; *P* = 0.0091) when general anesthesia was included as confounding factors, which did not change the main results of our study. In addition, further studies from other samples of patients with AIS are needed to validate our results (Alexandre et al., 2021a,b). Fourth, our study was designed to be cross-sectional; therefore, causality cannot be established. To compensate for this limitation, we performed a causal mediation analysis and suggested a possible association between eosinophils, SAP, and functional outcome (Figure 2; Figure V in Appendix A). Nevertheless, it is noteworthy that this is the first study showing the complex relationships among eosinophils, SAP, and functional outcome in patients with AIS experiencing mechanical thrombectomy. In this regard, attempts to maintain eosinophils have important implications for stroke outcomes, especially considering the clinical outcomes at 3 months being unsatisfactory with nearly half of the successfully reperfused patients experiencing unfavorable functional outcomes (Goyal et al., 2016). In addition, these results might eventually pave the way for finding out a suitable target for the prevention and treatment of SAP.

## Conclusion

This study shows that the decreased eosinophil level was associated with the high risk of SAP and poor functional outcome in patients with AIS experiencing mechanical thrombectomy, and SAP was in the path of the association between eosinophils and functional outcomes. These findings open new avenues of research on the complex relationship between eosinophils, SAP, and functional outcome. Further studies are needed to explore the role of eosinophils in SAP and its mechanism.

## Data Availability Statement

The raw data supporting the conclusions of this article will be made available by the authors, without undue reservation.

## Ethics Statement

The studies involving human participants were reviewed and approved by the Ethics Committee of the Second Affiliated Hospital of Soochow University. The patients/participants provided their written informed consent to participate in this study.

## Author Contributions

ZG, JH, ShuaY, GX, YC, and C-FL contributed to the concept and rationale for this study. ZG, JH, and ShuaY were responsible for the first draft. YC and ZG contributed to statistical analyses. ShuhY, HW, JX, HZ, SYou, and ZH performed the data collection and curation. GX, YC, and C-FL contributed to the first revision. All authors read and approved the final manuscript.

## Funding

This study was supported in part by the National Natural Science Foundation of China (Grant No. 81801154), the Suzhou City People's Livelihood Science and Technology Project (SYS2017051), and the Discipline Construction Program of the Second Affiliated Hospital of Soochow University (XKTJ-TD202004).

## Conflict of Interest

The authors declare that the research was conducted in the absence of any commercial or financial relationships that could be construed as a potential conflict of interest.

## Publisher's Note

All claims expressed in this article are solely those of the authors and do not necessarily represent those of their affiliated organizations, or those of the publisher, the editors and the reviewers. Any product that may be evaluated in this article, or claim that may be made by its manufacturer, is not guaranteed or endorsed by the publisher.

## Abbreviations

AIS: acute ischemic stroke
AF: atrial fibrillation
ASPECTS: Alberta Stroke Program Early CT Score
CI: confidence interval
IQR: interquartile range
IVT: intravenous thrombolysis
mRS: modified Rankin Scale
mTICI: modified Thrombolysis in Cerebral Infarction
NIHSS: National Institutes of Health Stroke Scale
OR: odds ratio
SAP: stroke-associated pneumonia.
