# Supplementary material for: Eosinophils, Stroke-Associated Pneumonia, and Outcome After Mechanical Thrombectomy for Acute Ischemic Stroke
Source: Front Aging Neurosci. 2022 May 23;14:830858. doi: 10.3389/fnagi.2022.830858 (PMC9168221; doi:10.3389/fnagi.2022.830858)
Supplement: Supplementary file 1 [file Data_Sheet_1.PDF]

Appendix A

Eosinophils, Stroke-associated Pneumonia, and Outcome after Mechanical Thrombectomy for Acute Ischemic Stroke

Table I. Demographic and clinical characteristics of included and excluded patients

Characteristics	Patients included	Patients excluded	*P* Value
No. of patients	328	69	
Age, y; median (IQR)	68.00 (57.00-75.00)	69.00 (55.00-76.00)	0.669
Female, n (%)	143 (43.60%)	22 (31.88%)	0.073
Atrial fibrillation, n (%)	147 (44.82%)	19 (27.54%)	0.008
Hypertension, n (%)	225 (68.60%)	55 (79.71%)	0.066
Diabetes, n (%)	61 (18.60%)	12(17.39%)	0.814
Hyperlipidemia, n (%)	115 (35.06%)	22(31.88%)	0.614
History of stroke, n (%)	53 (16.16%)	10 (14.49%)	0.731
Smoking, n (%)	100 (30.49%)	29 (42.03%)	0.063
Drinking, n (%)	73 (22.26%)	20 (28.99%)	0.230
Baseline NIHSS, median (IQR)	16.00 (13.00-20.00)	13.00 (10.00-19.00)	0.023
IVT, n (%)	103 (31.40%)	23 (33.33%)	0.754
Stroke etiology, n (%)			0.003
LAA	143 (43.60%)	43 (62.32%)	
Cardioembolic	168 (51.22%)	20 (28.99%)	
Others	17 (5.18%)	6 (8.70%)	
Premorbid mRS, median (IQR)	0.00 (0.00-0.00)	0.00 (0.00-0.00)	0.902

IQR, interquartile range; IVT, intravenous thrombolysis; mRS, modified Rankin Scale; NIHSS, National Institutes of Health Stroke Scale.

Table II. The collinearity screening of baseline characteristics

Characteristics	Variance inflation factor
Age	1.7
Female	1.7
Atrial fibrillation	2.1
Hypertension	1.3
Diabetes	1.1
Hyperlipidemia	1.1
History of stroke	1.8
Smoking	2
Drinking	1.7
Baseline NIHSS	1.2
ASPECTS	1.2
Occluded artery	1.2
IVT	1.1
Dysphagia	1.1
Premorbid mRS	1.8
Stroke etiology	1.8
Collateral score	1.3
OTR	1.2
Number of passes	1.2
mTICI score 2b or 3	1.1
Eosinophils	1.1

ASPECTS; Alberta Stroke Program Early CT Score; IVT, intravenous thrombolysis; mRS, modified Rankin Scale; mTICI, modified Thrombolysis in Cerebral Infarction; NIHSS, National Institutes of Health Stroke Scale; OTR, onset to reperfusion time.

We think collinearity exists and eliminate these covariables in the final models if their variance inflation factors are greater than or equal to 5.

Table III. Associations of covariates with stroke-associated pneumonia

Covariates	exp(beta)	95%CI	*P* Value
Age	1.0295	1.0125-1.0468	0.0006
Female	1.1617	0.7500-1.7993	0.5019
Atrial fibrillation	1.2138	0.7845-1.8781	0.3843
Hypertension	1.1209	0.7029-1.7875	0.6318
Diabetes	1.0853	0.6212-1.8960	0.7737
Hyperlipidemia	0.6753	0.4281-1.0650	0.0912
History of stroke	1.4694	0.8072-2.6749	0.2080
Smoking	1.0329	0.6450-1.6541	0.8928
Drinking	1.3027	0.7706-2.2023	0.3235
Baseline NIHSS	1.0464	1.0123-1.0816	0.0073
ASPECTS	0.7191	0.5405-0.9568	0.0236
Occluded artery			
ICA	Reference	Reference	Reference
M1 of the MCA	1.0101	0.5859-1.7416	0.9712
Posterior circulation	2.3636	1.0138-5.5106	0.0464
Others	1.0000	0.3836-2.6068	1.0000
IVT	0.7965	0.4993-1.2706	0.3396
Dysphagia	4.2414	2.6675-6.7439	<0.0001
Premorbid mRS	1.3331	0.8480-2.0955	0.2128
Stroke etiology			
LAA	Reference	Reference	Reference
Cardioembolic	0.9615	0.6146-1.5043	0.8636
Others	0.2564	0.0797-0.8245	0.0224
Collateral score	0.6835	0.5536-0.8439	0.0004

OTR	1.0001	0.9988-1.0014	0.8798
Number of passes	1.1718	0.9567-1.4354	0.1255
mTICI score 2b or 3	0.8339	0.4251-1.6356	0.5971

ASPECTS, Alberta Stroke Program Early CT Score; ICA, internal carotid artery; IVT, intravenous thrombolysis; MCA, middle cerebral artery; mRS, modified Rankin Scale; mTICI, modified Thrombolysis in Cerebral Infarction; NIHSS, National Institutes of Health Stroke Scale; OTR, onset to reperfusion time; Posterior circulation, including basilar artery and intracranial part of the vertebral artery; SAP, stroke-associated pneumonia.

Table IV. The adjusting roles of potential confounders on the estimates of eosinophils on stroke-associated pneumonia

+/- covariates	Basic model	Complete model	The selected covariates
Initial regression coefficient of eosinophils	-11.4899	-8.3709	
Age	-10.7463	-8.3542	
Female,	-11.3049	-8.3823	
Atrial fibrillation	-11.3464	-8.3769	
Hypertension	-11.5050	-8.6559	
Diabetes	-11.4837	-8.2284	
Hyperlipidemia	-11.1645	-8.2773	
History of stroke	-11.2378	-8.5356	
Smoking	-11.7941	-8.3154	
Drinking	-11.6869	-8.4567	
Baseline NIHSS	-10.0888 *	-8.6323	Yes
ASPECTS	-10.7396	-8.4599	
Occluded artery	-11.5969	-7.9063	
IVT	-11.3862	-8.2983	
Dysphagia	-8.7436 *	-9.6963 *	Yes
Premorbid mRS	-11.3129	-8.3188	
Stroke etiology	-12.0757	-7.7516	
Collateral score	-10.5156	-8.4927	
OTR	-11.4985	-8.6086	
Number of passes	-11.1274	-8.5042	
mTICI score 2b or 3	-11.4026	-8.4357	

* These confounders changed the estimates of eosinophils on stroke-associated pneumonia score by more than 10% when introduce covariates into the basic model or remove covariates from the complete model.

Table V. The selected covariates when eosinophils as a continuous variable

Y	X	The selected covariates （Criterion 1）	The selected covariates（Criterion 2）
Stroke-associated pneumonia	Eosinophils	Baseline NIHSS, Dysphagia	Age, Hyperlipidemia, Baseline NIHSS, ASPECTS, Occluded artery, Dysphagia, Stroke etiology, Collateral score
mRS score	Eosinophils	Baseline NIHSS, ASPECTS, Dysphagia, Collateral score	Age, Female, Atrial fibrillation, Hypertension, Diabetes, History of stroke, Smoking, Drinking, Baseline NIHSS, ASPECTS, Occluded artery, Dysphagia, Collateral score, OTR, mTICI score 2b or 3
Poor outcome	Eosinophils	Baseline NIHSS, Dysphagia	Age, Female, Atrial fibrillation, Hypertension, Diabetes, History of stroke, Smoking, Drinking, Baseline NIHSS, ASPECTS, Occluded artery, Dysphagia, Collateral score, Number of passes, mTICI score 2b or 3

Criterion 1: These confounders changed the estimates of eosinophils on the outcomes of interest by more than 10% when introduce covariates into the basic model or remove covariates from the complete model.

Criterion 2: These variables were significantly associated with stroke-associated pneumonia score ($P < 0.10$) or changed the estimates of eosinophils on the outcomes of interest by more than 10%.

Table VI. The selected covariates when eosinophils as a categorical variable (dichotomous)

Y	X	The selected covariates （Criterion 1）	The selected covariates （Criterion 2）
Stroke-associated pneumonia	Eosinophils (dichotomous)	Baseline NIHSS, Occluded artery, Dysphagia, Collateral score	Age, Hyperlipidemia, Baseline NIHSS, ASPECTS, Occluded artery, Dysphagia, Stroke etiology, Collateral score
mRS score	Eosinophils (dichotomous)	Baseline NIHSS, ASPECTS, Dysphagia, Collateral score	Age, Female, Atrial fibrillation, Hypertension, Diabetes, History of stroke, Smoking, Drinking, Baseline NIHSS, ASPECTS, Occluded artery, Dysphagia, Collateral score, OTR, mTICI score 2b or 3
Poor outcome	Eosinophils (dichotomous)	Baseline NIHSS	Age, Female, Atrial fibrillation, Hypertension, Diabetes, History of stroke, Smoking, Drinking, Baseline NIHSS, ASPECTS, Occluded artery, Dysphagia, Collateral score, Number of passes, mTICI score 2b or 3

Criterion 1: These confounders changed the estimates of eosinophils on the outcomes of interest by more than 10% when introduce covariates into the basic model or remove covariates from the complete model.

Criterion 2: These variables were significantly associated with stroke-associated pneumonia score ($P < 0.10$) or changed the estimates of eosinophils on the outcomes of interest by more than 10%.

Table VII. Relationship between eosinophils (dichotomous) as a categorical variable and the SAP/functional outcome among patients with acute ischemic stroke in different models

Variable	Non-adjusted model		Model 1		Model 2	
	β/ OR (95%CI)	*P* Value	β/ OR (95%CI)	*P* Value	β/ OR (95%CI)	*P* Value
SAP	0.40 (0.25, 0.63)	<0.0001	0.40 (0.25, 0.64)	0.0001	0.54 (0.31, 0.96)	0.0342
mRS	-1.63 (-2.04, -1.22)	<0.0001	-1.51 (-1.91, -1.12)	<0.0001	-0.80 (-1.15, -0.44)	<0.0001
Poor outcome (mRS score 3-6)	0.18 (0.10, 0.31)	<0.0001	0.17 (0.10, 0.30)	<0.0001	0.20 (0.09, 0.45)	0.0001

Non-adjusted model: we did not adjust other covariates.

Model 1: we adjusted age and sex.

Model 2: we adjusted variables which were significantly associated with outcomes of interest ($p < 0.10$) or changed the estimates of eosinophils on outcomes of interest by more than 10% .

mRS, modified Rankin Scale; OR, odds ratio; SAP, stroke-associated pneumonia.

Table VIII. Subgroup analyses on the association between eosinophils and stroke-associated pneumonia among patients with acute ischemic stroke experiencing mechanical thrombectomy

Subgroup	N	OR (95%CI)	*P* Value	*P* for interaction
Age, years				0. 4979
＜65	135	0.01 (0.00, 638.78)	0.3977	
≥65	193	.00 (0.00, 16.73)	0.14097	
Sex				0.0705
Male	185	0.00 (0.00, 1.58)	0.0608	
Female	143	0.00 (0.00, 0.15)	0.0277	
Atrial fibrillation				
No	181	0.00 (0.00, 1.39)	0.0585	
Yes	147	0.00 (0.00, 1.88)	0.0579	
Hypertension				0.2521
No	103	0.00 (0.00, 478.81)	0.1738	
Yes	225	0.00 (0.00, 3.78)	0.0941	
Diabetes				0.1893
No	267	0.00 (0.00, 0.30)	0.0242	
Yes	61	0.00 (0.00, Inf)	0.9998	
Hyperlipidemia				0.9528
No	213	0.03 (0.00, 167.87)	0.4381	
Yes	115	0.00 (0.00, 0.00)	0.0050	
History of stroke				
No	275	0.00 (0.00, 0.53)	0.0336	
Yes	53	0.00 (0.00, Inf)	0.9999	
Smoking				0.3149

No	228	0.00 (0.00, 3.86)	0.0937	
Yes	100	0.00 (0.00, 1.39)	0.0548	
Drinking				0.3013
No	255	0.00 (0.00, 0.21)	0.0207	
Yes	73	0.00 (0.00, inf.)	0.2172	
Baseline NIHSS				0.2513
＜16	151	0.00 (0.00, 0.01)	0.0068	
≥16	177	0.02 (0.00, 1099.30)	0.4896	
IVT				0.4714
No	225	0.00 (0.00, 12.71)	0.1433	
Yes	103	0.00 (0.00, 7.46)	0.0782	
Dysphagia				0.4207
No	147	0.00 (0.00, 0.00)	0.0047	
Yes	181	0.92 (0.00, 437362.15)	0.9902	

In the multivariate models, confounding factors, such as age, hyperlipidemia, baseline NIHSS, ASPECTS, occluded artery, dysphagia, stroke etiology and ollateral score were included unless the variable was used as a stratification variable.

IVT, intravenous thrombolysis; N, No. of participants; NIHSS, National Institutes of Health Stroke Scale; OR, odds ratio.

Table IX. Subgroup analyses on the association between eosinophils and functional outcome

Subgroup	N	mRS			Poor outcome (mRS score 3-6)		
		β(95%CI)	*P* value	*P* for interaction	OR (95%CI)	*P* value	*P* for interaction
Age, years				0.6399			0.9779
＜65	135	-7.26 (-13.36, -1.17)	0.0216		0.00 (0.00, 36.90)	0.1319	
≥65	193	-3.68 (-9.16, 1.80)	0.1902		0.00 (0.00, 1.65)	0.0637	
Sex				0.8170			0.6670
Male	185	-8.24 (-13.55, -2.94)	0.0027		0.00 (0.00, 0.04)	0.0164	
Female	143	-4.20 (-10.79, 2.38)	0.2136		0.00 (0.00, 3.78)	0.0725	
Atrial fibrillation				0.5692			0.1556
No	181	-4.59 (-9.13, -0.05)	0.0494		0.00 (0.00, 23.20)	0.1896	
Yes	147	-5.99 (-14.85,	0.1882		0.00 (0.00, 0.27)	0.0379	

		2.87)					
Hypertension				0.1596			0.8406
No	103	-2.32 (-8.53, 3.90)	0.4677		0.00 (0.00, inf.)	0.6302	
Yes	225	-7.82 (-13.31, -2.32)	0.0059		0.00 (0.00, 0.02)	0.0062	
Diabetes				0.4900			0.3731
No	267	-7.28 (-11.63, -2.93)	0.0012		0.00 (0.00, 0.02)	0.0055	
Yes	61	2.15 (-11.04, 15.33)	0.7521		inf. (0.00, Inf)	1.0000	
Hyperlipidemia				0.1555			0.0590
No	213	-0.79 (-5.70, 4.11)	0.7519		0.04 (0.00, 451.32)	0.4981	
Yes	115	-9.98 (-17.34, -2.61)	0.0095		0.00 (0.00, 0.48)	0.0460	
History of stroke				0.5344			0.5457
No	275	-6.91 (-11.47,	0.0033		0.00 (0.00, 0.04)	0.0070	

		-2.35)					
Yes	53	4.82 (-4.68, 14.33)	0.3318		inf. (0.00, Inf)	1.0000	
Smoking				0.9225			0.5764
No	228	-2.94 (-7.47, 1.60)	0.2055		0.00 (0.00, 7.82)	0.1175	
Yes	100	-16.17 (-24.50, -7.84)	0.0003		0.00 (0.00, 0.00)	0.0100	
Drinking				0.4709			0.1983
No	255	-4.94 (-9.23, -0.66)	0.0248		0.00 (0.00, 0.09)	0.0127	
Yes	73	-13.41 (-23.19, -3.63)	0.0103		0.00 (0.00, Inf)	0.9997	
Baseline NIHSS				0.7061			0.8105
＜16	151	-6.63 (-12.08, -1.18)	0.0187		0.00 (0.00, 0.22)	0.0270	
≥16	177	-5.19 (-12.04, 1.65)	0.1393		0.00 (0.00, 0.89)	0.0480	
IVT				0.2595			0.1412

No	225	-5.32 (-11.13, 0.50)	0.0750		0.00 (0.00, 22.26)	0.1900	
Yes	103	-5.14 (-10.81, 0.52)	0.0797		0.00 (0.00, Inf)	0.9602	
Dysphagia				0.1736			0.1441
No	147	-4.94 (-9.76, -0.12)	0.0471		0.00 (0.00, 0.29)	0.0315	
Yes	181	-7.64 (-15.67, 0.40)	0.0645		0.00 (0.00, 0.34)	0.0361	

In the multivariate models, confounding factors were included unless the variable was used as a stratification variable.

IVT, intravenous thrombolysis; N, No. of participants; NIHSS, National Institutes of Health Stroke Scale; OR, odds ratio.

Table X. Association of eosinophils with poor outcome was independent of SAP

Exposure	Non-adjusted	Model 1	Model 2
	β/ OR; (95%CI); *P* Value	β/ OR; (95%CI); *P* Value	β/ OR; (95%CI); *P* Value
mRS			
Eosinophils	-12.43 (-17.26, -7.60) <0.0001	-11.30 (-15.89, -6.71) <0.0001	-4.87 (-8.77, -0.97) 0.0150
Eosinophils (dichotomous)			
Low eosinophil level	0	0	0
High eosinophil level	-1.63 (-2.04, -1.22) <0.0001	-1.51 (-1.91, -1.12) <0.0001	-0.76 (-1.11, -0.40) <0.0001
Poor outcome (mRS score 3-6)			
Eosinophils	0.00 (0.00, 0.00) <0.0001	0.00 (0.00, 0.00) 0.0001	0.00 (0.00, 0.39) 0.0269
Eosinophils (dichotomous)			
Low eosinophil level	1.0	1.0	1.0
High eosinophil level	0.18 (0.10, 0.31) <0.0001	0.17 (0.10, 0.30) <0.0001	0.21 (0.09, 0.49) 0.0003

Non-adjusted model: we did not adjust other covariates.

Model 1: we adjusted age and sex.

Model 2: we adjusted variables which were significantly associated with outcomes of interest ($p < 0.10$) or changed the estimates of eosinophils on outcomes of

interest by more than 10%.

mRS, modified Rankin Scale; OR, odds ratio; SAP, stroke-associated pneumonia.

Figure I. Flow chart of patient cohort

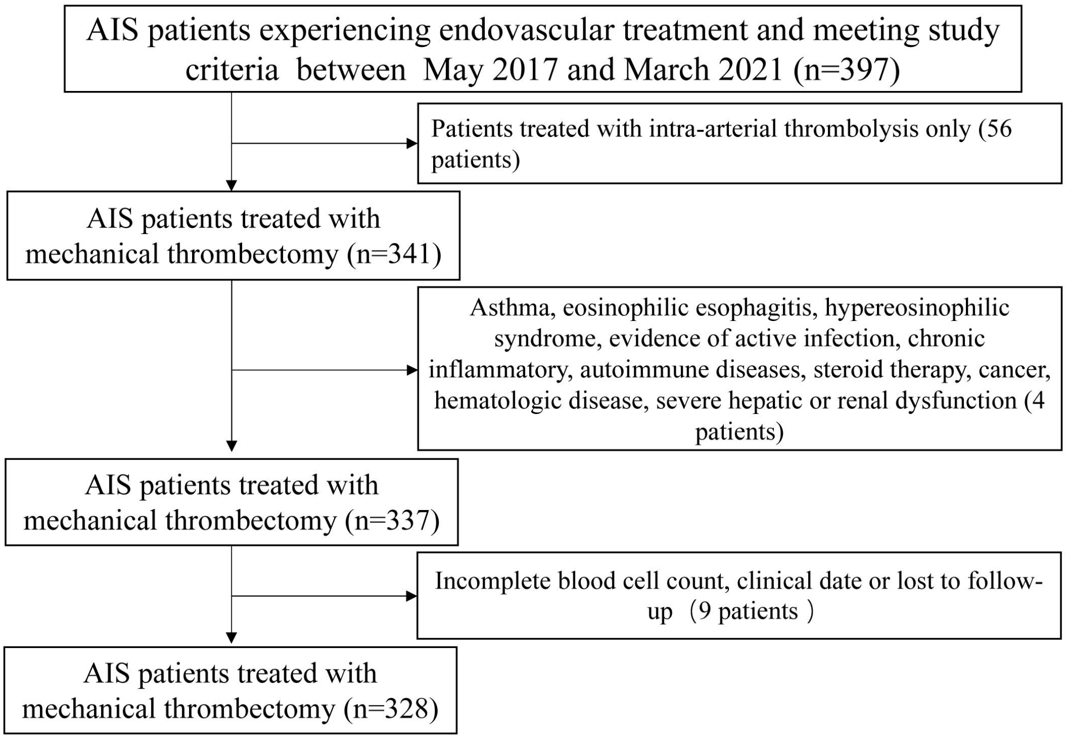

AIS: Acute ischemic stroke.

Fig II. The typical imaging manifestations of SAP on the CT

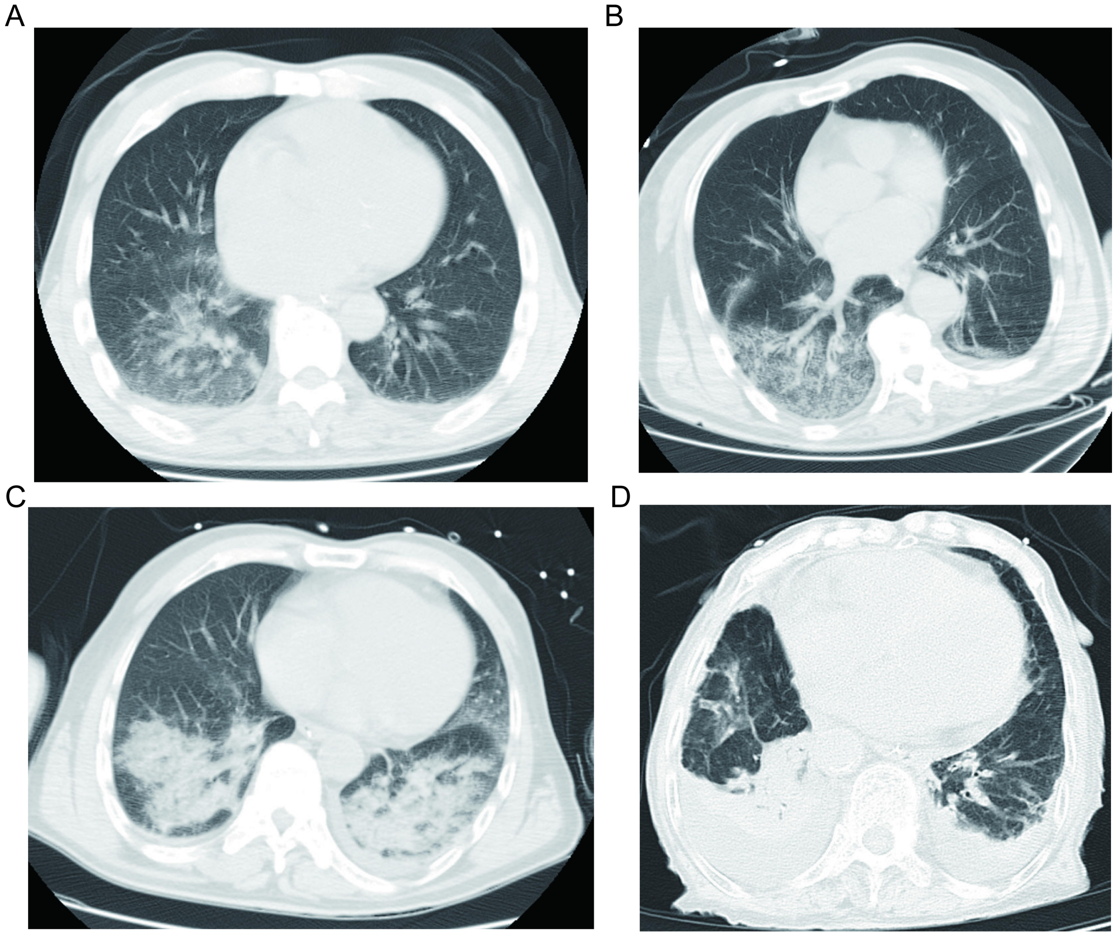

The chest CT findings in patients with stroke-associated pneumonia are diverse, including bronchial wall thickening, nodules, consolidation, pleural effusion, bronchiectasis, groundglass attenuation, and atelectasis; however, the location of pulmonary findings were more prevalently distributed in the right lung and in lower lung zones on the CT, suggestive for aspiration.

Fig III. A non-linear relationship was detected between eosinophils and mRS score in patients with acute ischemic stroke experiencing mechanical thrombectomy

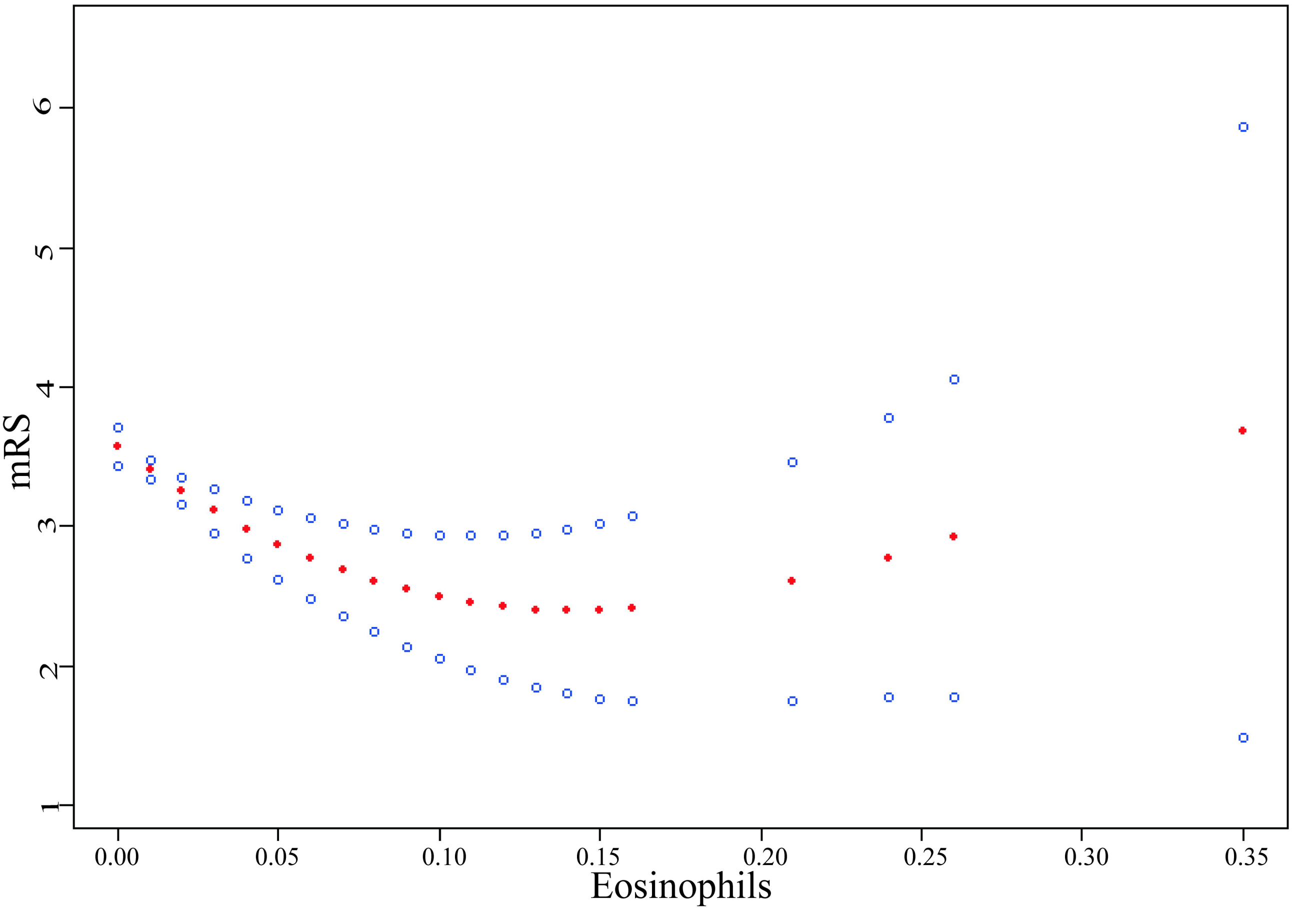

A nonlinear relationship between them was detected after adjusting for age, female, atrial fibrillation, hypertension, diabetes, history of stroke, smoking, drinking, baseline NIHSS, ASPECTS, occluded artery, dysphagia, collateral score, OTR and mTICI score 2b or 3.

mRS, modified Rankin Scale.

Fig IV. A non-linear relationship was detected between eosinophils and poor outcome in patients with acute ischemic stroke experiencing mechanical thrombectomy

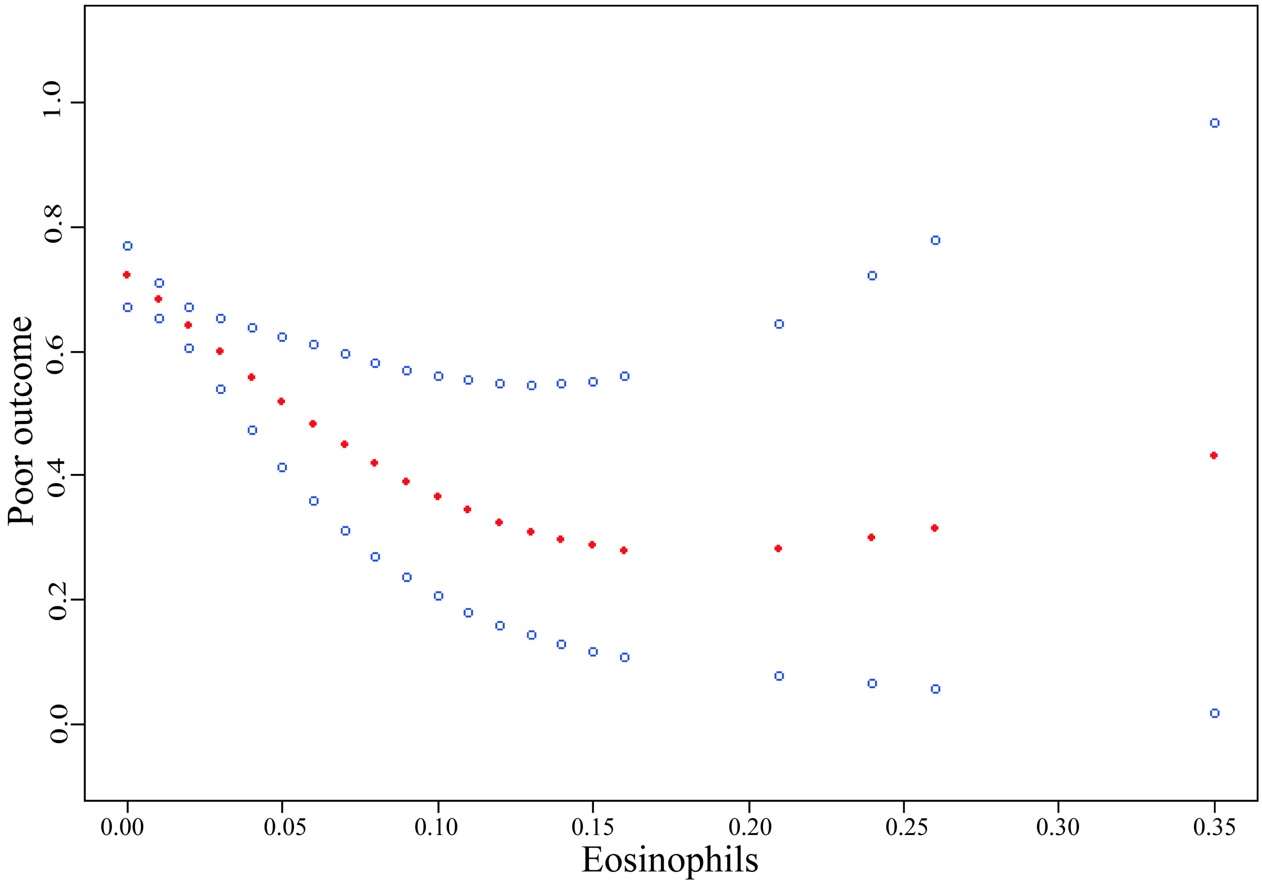

A nonlinear relationship between them was detected after adjusting for age, female, atrial fibrillation, hypertension, diabetes, history of stroke, smoking, drinking, baseline NIHSS, ASPECTS, occluded artery, dysphagia, collateral score, number of passes, mTICI score 2b or 3.

Fig V. Schematic diagram of mediation analyses for mRS score

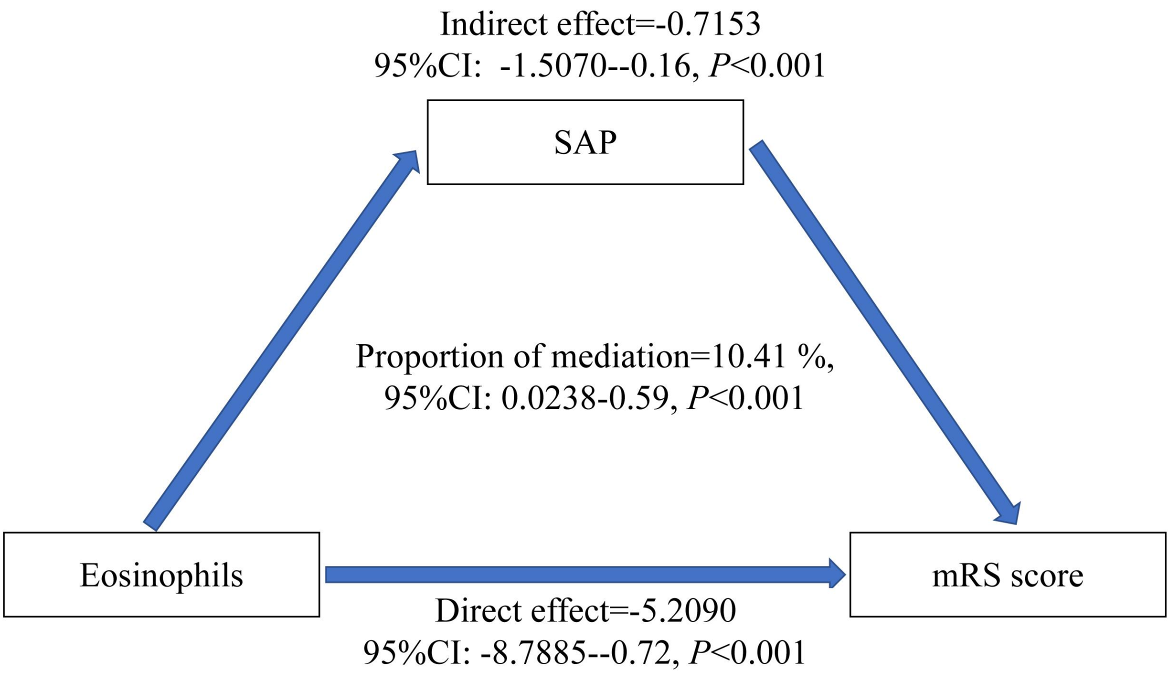

The eosinophils were entered as predictors. stroke-associated pneumonia score was entered as a mediator. The proportion of the total effect of eosinophils on the mRS score mediated by stroke-associated pneumonia was 10.41% (95% CI, 2.38%–59%). The direct effect of eosinophils on the mRS score (total effect minus indirect effect) was still statistically significant (P<0.001) after removing the effect mediated by stroke-associated pneumonia.

mRS, modified Rankin Scale.

Fig VI. Schematic diagram of the relationships between eosinophils, SAP, neuroprotection, and clinical outcome after mechanical thrombectomy in patients with acute ischemic stroke

.

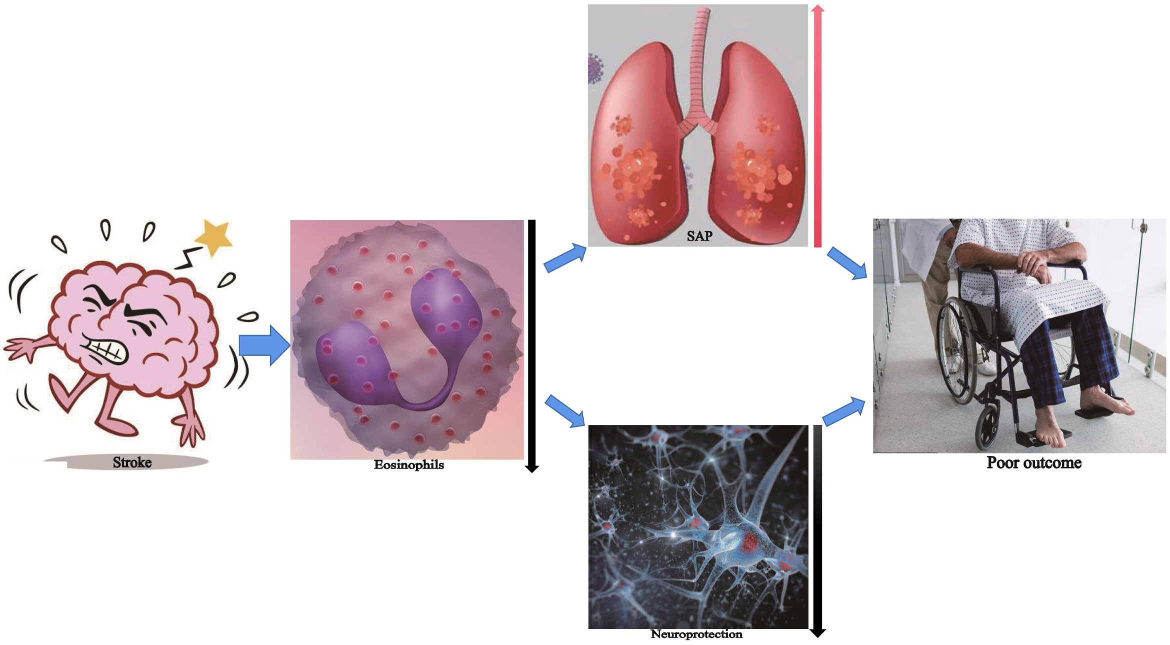

This schematic diagram is still the only hypothesis to reveal the relationships between eosinophils, SAP, neuroprotection, and clinical outcome: stroke triggers an acute decrease in circulating eosinophil counts, and decreased eosinophils may contribute to more SAP and less neuroprotection, which in turn lead to poor outcome.

SAP, stroke-associated pneumonia.
